# Risk of Micronutrient Inadequacy among Hispanic, Lactating Mothers: Preliminary Evidence from the Southern California Mother’s Milk Study

**DOI:** 10.3390/nu13093252

**Published:** 2021-09-18

**Authors:** Laura E. Wild, William B. Patterson, Roshonda B. Jones, Jasmine F. Plows, Paige K. Berger, Claudia Rios, Jennifer L. Fogel, Michael I. Goran, Tanya L. Alderete

**Affiliations:** 1 Department of Integrative Physiology, University of Colorado Boulder, 354 UCB, Boulder, CO 80309, USA; laura.wild@colorado.edu (L.E.W.); william.patterson-1@colorado.edu (W.B.P.); 2 Department of Pediatrics, The Saban Research Institute, Children’s Hospital Los Angeles, University of Southern California, 4650 Sunset Boulevard, Los Angeles, CA 90027, USA; rbarnerjones@gmail.com (R.B.J.); jplows@chla.usc.edu (J.F.P.); paberger@chla.usc.edu (P.K.B.); clrios@chla.usc.edu (C.R.); jfogel@chla.usc.edu (J.L.F.); mgoran@chla.usc.edu (M.I.G.)

**Keywords:** nutrient inadequacy, postpartum diet, micronutrients, lactating women, Hispanics

## Abstract

Micronutrients are dietary components important for health and physiological function, and inadequate intake of these nutrients can contribute to poor health outcomes. The risk of inadequate micronutrient intake has been shown to be greater among low-income Hispanics and postpartum and lactating women. Therefore, we aimed to determine the risk of nutrient inadequacies based on preliminary evidence among postpartum, Hispanic women. Risk of micronutrient inadequacy for Hispanic women (29–45 years of age) from the Southern California Mother’s Milk Study (*n* = 188) was assessed using 24 h dietary recalls at 1 and 6 months postpartum and the estimated average requirement (EAR) fixed cut-point approach. Women were considered at risk of inadequate intake for a nutrient if more than 50% of women were consuming below the EAR. The Chronic Disease Risk Reduction (CDRR) value was also used to assess sodium intake. These women were at risk of inadequate intake for folate and vitamins A, D, and E, with 87.0%, 93.4%, 43.8%, and 95% of women consuming less than the EAR for these nutrients, respectively. Lastly, 71.7% of women consumed excess sodium. Results from this preliminary analysis indicate that Hispanic women are at risk of inadequate intake of important micronutrients for maternal and child health.

## 1. Introduction

Micronutrient intake is essential for optimal physiological function. Approximately 2 billion people worldwide are affected by micronutrient deficiencies, which occur predominately within developing countries and are more common among pregnant women and infants under the age of five due to the increase in physiological demand for micronutrients by the fetus during pregnancy and the nursing infant during lactation [1]. In more developed countries, such as the United States, however, micronutrient inadequacies are more prevalent, defined as consuming less than the recommended intake of individual nutrients specific for age, sex, and life-stage group [2]. This is of concern as the average American diet is high in energy-dense, nutrient-poor foods [3] and approximately 80% of the American public does not consume the recommended intake of nutrient-dense foods such as fruits and vegetables [4]. Inadequate consumption of these micronutrients may increase the risk of developing chronic diseases including obesity and type 2 diabetes [5,6] as well as contribute to fatigue [7] and diminished immune and cognitive function [6]. 

Poverty is one of the main contributing factors to inadequate micronutrient intake as this may potentiate food insecurity and limited access to nutritious foods [1,8,9,10]. In the United States, approximately 18.5% of Hispanic households are food insecure compared to 12.3% of all American households [11]. As a result, Hispanics have higher frequencies of methyl-nutrient and antioxidant inadequacies compared to non-Hispanic whites [12]. Of further concern is that pregnancy and the postpartum period are sensitive times for nutrient inadequacies [1,13] and food insecurity [14,15], where nutrient inadequacies have been associated with greater postpartum weight [16], increased stress and postpartum mental disorders [15,16,17,18], and poorer infant and child health outcomes [1,17,18,19,20]. In addition, nutrient demands increase for lactating women [21], and inadequate nutrition during lactation has been associated with decreased concentration of specific nutrients in breastmilk [22,23]. 

Despite the risks observed among the Hispanic population and postpartum and lactating women, no studies have yet assessed the risk of micronutrient inadequacies among lactating, Hispanic women. Therefore, the aim of this study was to determine the risk of micronutrient inadequacy based on preliminary evidence among Hispanic, lactating women for vitamins (i.e., folate, niacin, pantothenic acid, riboflavin, thiamin, vitamin A, vitamin B6, vitamin B12, vitamin C, vitamin D, vitamin E, and vitamin K) and minerals (i.e., calcium, copper, iron, magnesium, manganese, phosphorus, potassium, selenium, sodium, and zinc). Secondarily, we aimed to determine whether these participants were consuming excessive sodium, which may contribute to chronic disease.

## 2. Materials and Methods

### 2.1. Study Participants

Participants in this study were recruited between 2016 and 2020 for the Southern California Mother’s Milk Study (NIH R01 DK110793). The Mother’s Milk Study is an ongoing longitudinal cohort study that is investigating breast milk factors and the gut microbiota during the first two years postpartum in 240 Hispanic mother–infant pairs [24]. Mother–infant dyads are being selected from maternity clinics affiliated with the University of Southern California in Los Angeles County. At the time of our analysis, 222 participants had completed at least one clinical visit. Of these women, 188 had complete dietary intake information in the first 6 months postpartum. 

We included women who self-identified as Hispanic ethnicity; were greater than 18 years old at the time of delivery; had a healthy, term, singleton birth; were within 1 month postpartum; and planned to breastfeed for at least three months postpartum. Participants using medications or with any medical conditions affecting metabolism, nutritional status, or physical or mental health were excluded from this study. In addition, we excluded participants using tobacco products (i.e., >1 cigarette per week) or recreational drugs as well as those that had a clinical diagnosis of fetal abnormalities. Written informed consent was obtained from participants prior to enrollment and the Institutional Review Boards (IRB) at the University of Southern California (USC), Children’s Hospital Los Angeles (CHLA), and University of Colorado Boulder (CU Boulder) approved this study.

### 2.2. Study Visits

Participants in the Mother’s Milk Study completed one clinical visit at 1 month postpartum as well as a clinical visit at 6 months postpartum. At each of these visits, information regarding family and maternal health was collected using questionnaires [24]. Information regarding breastfeeding practices was also collected using questionnaires from which average breast feedings per day from the prior 7 days were scored as never, less than once per day, 1–8 times per day, or greater than 8 times per day [25]. Previous studies have determined that women are predominately breastfeeding if at least 80% of infant feedings are from breastmilk [26]. Based on this, we classified women in the current study as primarily breastfeeding if they had an average of 7 or more breast feedings per day. Additionally, maternal weight (kg) was measured using an electronic scale (Tanita Bc-549 Plus Ironman Body Composition Monitor, Tanita, Tokyo, Japan) and standing height (m) was measured using a stadiometer (Seca 126, Seca GmBH & Co. KG, Hamburg, Germany) to calculate maternal body mass index (BMI, kg/m^2^). Maternal pre-pregnancy BMI and age at delivery were also collected at the first clinical visit. Maternal BMI was used to categorize mothers as normal weight, overweight, or obese based on the Center for Disease Control and Prevention (CDC) classifications [27]. Finally, information regarding parental education and occupation was collected using a questionnaire at the first clinical visit to calculate individual socioeconomic status (SES) based on a modified version of the four-factor Hollingshead Index [28], where students, stay-at-home parents, and unemployed persons were assigned a score of zero to keep them in the analysis [29]. The Hollingshead Index calculates SES with a range of scores from 8 to 66 with higher scores indicating higher SES. 

### 2.3. Dietary Intake

Two, non-consecutive, 24 h dietary recalls were performed as part of the 1 and 6 month postpartum visits for a total of four recalls per participant. At each timepoint, every participant had two recalls, with one detailing intake during a weekday and the other detailing intake during a weekend to represent dietary intake throughout the week. Dietary recalls were used to assess diet including total energy intake, total macronutrient intake (i.e., carbohydrate, protein, and fat), vitamin intake (i.e., folate, niacin, pantothenic acid, riboflavin, thiamine, vitamin A, vitamin B6, vitamin B12, vitamin C, vitamin D, vitamin E, and vitamin K), and mineral intake (i.e., calcium, copper, iron, magnesium, manganese, phosphorus, potassium, selenium, sodium, and zinc). Trained research staff conducted the dietary recalls using the multi-pass method [30]. Dietary supplement information was also assessed during the 24 h dietary recalls, where details were obtained on the participants’ use of vitamins, minerals, herbal supplements, and other supplements over the previous 30 days, including whether it was taken on the day of intake being assessed. Information regarding supplement type, consumption frequency, and amount taken was also recorded. The first recall was performed in person with the use of food models, portion booklets, or serving containers to assist in estimating serving sizes. The remaining recalls were conducted by telephone. Dietary data were analyzed using Nutrition Data System for Research (NDSR) software (version 2018).

### 2.4. Statistical Analysis

The 24 h dietary recall is used to determine intake on a given day; however, this method may not provide reliable estimates of usual nutrient intakes due to high within-person variability [13,31,32,33]. For this reason, macros developed to implement the National Cancer Institute (NCI) method were used to produce the average usual intake, as well as the population prevalence that were below the estimated average requirement (EAR) or the adequate intake (AI) using the cut-point approach [34]. In the current study, EAR values used were specific to lactating women to determine the proportion of women meeting these guidelines since 98.4% and 81.4% women were breastfeeding at 1 and 6 months postpartum, respectively. The cut-point method, which we used for all nutrients, including iron, provides an estimate of the proportion of individuals in the study population that are at risk of inadequate intakes [13]. For nutrients without an established EAR (i.e., pantothenic acid, vitamin K, manganese, and potassium), we assessed the percentage of women with usual intake below the AI using the same cut-point method. Briefly, the average usual intake and distribution for each micronutrient was calculated using information from all dietary recalls (i.e., two at 1month postpartum and two at 6 months postpartum). Covariates for usual intake determination included timepoint (i.e., 1 month, 6 months postpartum) and day of the week of the 24 h recall (i.e., weekday [Monday–Thursday] and weekend day [Friday–Sunday]). Average maternal weight increased over the first 6 months postpartum; therefore, sensitivity analyses were performed by also including maternal weight as a covariate, but results were unchanged (data not shown). We considered women to be at risk of inadequate intake for a nutrient if fewer than 50% of women were meeting the EAR value. Results were largely unchanged when examining maternal diet separately at 1 and 6 months postpartum (Supplemental Table S1). In addition, we assessed the number of women exceeding the Chronic Disease Risk Reduction (CDRR) intake for sodium for lactating women, which is the estimated intake value of sodium that would be expected to reduce chronic disease risk within a health population of the same age, sex, and life-stage group [35]. Lastly, differences in maternal weight and BMI were determined using paired *t*-tests, and statistical significance was considered *p* < 0.05. All analyses were performed in R (Version 4.0.2) and SAS (Version 9.4). 

## 3. Results

Among the 188 women included in this study, the average age of the participants was 29 years (range 18–45 years), and approximately 60% (*n* = 111) were of a low SES as indicated by an average Hollingshead Index composite score of 26.6 (SD = 11.9). At 1month postpartum, 98.4% (*n* = 185) of women were breastfeeding, with 64.9% (*n* = 120) of these women primarily breastfeeding their infants. Further, of the 81.4% (*n* = 153) of women breastfeeding at 6 months postpartum, 30.7% (*n* = 47) of these women continued to primarily breastfeed. In addition, at 1 month postpartum, women had an average weight of 75.1 kg (SD = 13.9 kg) and were predominately overweight and obese with a mean BMI of 30.2 kg/m^2^ (SD = 5.1 kg/m^2^). At 6 months postpartum, the average maternal weight increased to 76.7 kg (SD = 15.2 kg; *p*-value < 0.001), and the average BMI increased to 30.9 kg/m^2^ (SD = 5.7 kg/m^2^; *p*-value < 0.001).

### Risk of Micronutrient Inadequacies and Chronic Disease Reduction Risk 

**Figure 1:**
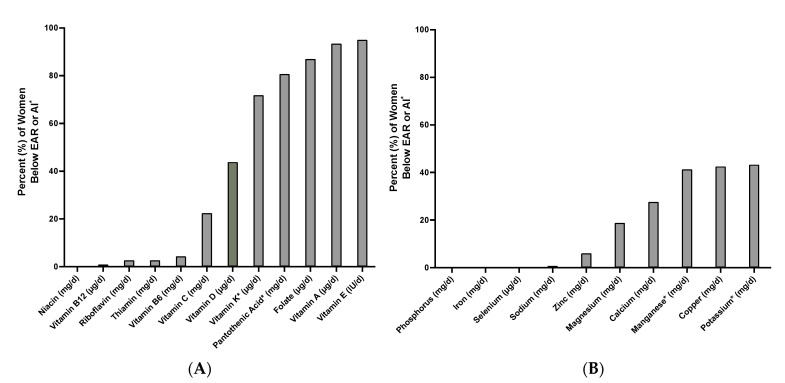
Percent of Hispanic women (*n* = 188) below the estimated average requirement (EAR) or adequate intake (AI) values for (**A**) vitamins and (**B**) minerals from 1 to 6 months postpartum. EAR and AI values come from the National Institutes of Health (NIH) for lactating women 19–50 years old. * EAR values have not been established for these nutrients, so adequate intake (AI) values were used.

Average usual intake after adjusting for within-person variation using the NCI method [36] is shown in Table 1. Of the 22 micronutrients examined, the postpartum Hispanic women included in this study were at risk of inadequate intake for three vitamins (Table 1 and Figure 1). Specifically, the average intake of folate was 355.6 μg/day (296.7–405.0) and 87.0% of women were not meeting the EAR value of 450 μg/day from 1 to 6 months postpartum. Women were also at risk of inadequate intake for vitamins A and E, with an average intake of 629.2 μg/day (509.1–732.5) and 12.9 IU/day (8.8–15.7) and 93.4% and 95% of women consuming less than the EAR values of 900 μg/day and 23.9 IU/day for these vitamins, respectively. In addition, while 43.8% of women did not meet the EAR value of 10 μg/day for vitamin D, the average intake was 11.8 μg/day (7.6–14.9). Lastly, the average adjusted intake of sodium was 2687.2 mg/day (245.8–3053.0). Thus, in the current study, approximately 71.7% of women exceeded the CDRR intake for sodium, which is less than 2300 mg/day for lactating women aged 19–50 years.

## 4. Discussion

Micronutrients are important dietary components, and adequate intake of these nutrients may protect against chronic disease [5,6], fatigue [7], and diminished immune and cognitive function [6]. Previous studies have shown that adequate intake of micronutrients is particularly important for lactating women [14,15,21], and Hispanics are at an increased risk of vitamin and mineral inadequacies [12,37]. Despite this, few studies have examined the risk for micronutrient inadequacies among this group. To our knowledge, this is the first study to examine preliminary evidence regarding the risk of micronutrient inadequacy among postpartum, lactating Hispanic women in the first 6 months postpartum. Results from this study indicate that Hispanic women during the postpartum period were at risk of inadequate intake for several vitamins. In addition, our results indicate that over half of these participants consumed more than the CDRR value for sodium.

Our results indicate that Hispanic, postpartum women were at risk of inadequate intake of folate with 87.0% consuming below the EAR for this nutrient. Importantly, inadequate folate intake may contribute to increased plasma homocysteine levels, which has been associated with increased risk of developing chronic vascular disease [38], dementia and Alzheimer’s disease [39], certain cancers [40], as well as decreased cognitive function [39]. Of particular concern in the present study is the high prevalence of women at risk of inadequate folate intake shortly after pregnancy as it is well established that folate deficiency during pregnancy can cause significant fetal neural tube defects [41]. 

We also found that most of the participants were at risk of inadequate intake of vitamin A with 93.4% of these women consuming below the recommended intake of this nutrient. Vitamin A is a fat-soluble vitamin important for optimal eye health and vision [42] and maintaining epithelial barriers (e.g., intestinal, lungs) [43] as well as acts as an anti-inflammatory agent [44]. Inadequate intake of vitamin A during lactation has also been associated with decreased breastmilk concentration of retinol, a vitamin A derivative [22]. This is an important consideration for infant health as increased intake of vitamin A among children has been associated with decreased risk of pediatric stunting [45,46] as well as xerophthalmia [47]. In addition to vitamin A, women were nearly at risk of inadequate intake of vitamin D with 43.8% of women consuming below the EAR value for this vitamin. Vitamin D is predominately synthesized intradermally with exposure to ultraviolet B radiation [48]. Vitamin D contributes to calcium absorption and homeostasis, and inadequate intake is associated with increased risk of developing osteoporosis [48]. This is particularly important for women, who have greater rates of osteoporosis than men [49]. Vitamin D is also important for optimal immune function and lower vitamin D intake has been associated with increased rates of infection and autoimmune diseases [50]. Lastly, lower serum vitamin D concentrations have been linked with obesity [51], and the women in our study were largely overweight or obese at both 1 and 6 months postpartum. 

This study also found that the women were at risk of inadequate intake of vitamin E as 95% of our participants had average consumption below the adequate intake of this vitamin. Vitamin E is also a fat-soluble vitamin and is a powerful antioxidant that works to stabilize reactive oxygen species and, therefore, prevent stress-induced DNA damage [52,53]. In addition, vitamin E may also be protective against Alzheimer’s disease as vitamin E may inhibit the production of hydrogen peroxide [53], which is a known factor in the development of this disease [54]. Finally, we determined that 71.7% of our participants consumed more than the CDRR value for sodium. It is well established that increased sodium intake is associated with increased risk of developing hypertension [55,56,57], particularly in the setting of low potassium intake [58,59]. In addition, it is also known that lactating women consume greater amounts of sodium in comparison to non-lactating women of the same age, which is in part due to increased food consumption during this time [35].

To our knowledge, this is the first study to examine preliminary evidence regarding the risk of micronutrient inadequacies among Hispanic women during a critical time that is characterized by increased nutritional demands. Despite these strengths, this study is limited by the exclusion of other racial/ethnic groups. In addition, our study is limited by a small sample size. Subsequent studies should include a more diverse and larger sample of postpartum women to better generalize these results to a greater population of women. Moreover, although we used multiple 24 h dietary recalls and employed the multi-pass method, dietary recalls are often subject to bias and are dependent on participants’ memory [60]. Additional studies should consider further dietary evaluations to fully apprehend maternal dietary patterns during this time. Lastly, future studies should also examine the risk prevalence and impact of these dietary variables during the pregnancy period since pregnant women are at high risk for micronutrient deficiencies and inadequacies [1,13]. This could include examining how gravidity and parity affect risk of micronutrient inadequacies for women. In addition, studies could correlate micronutrient intake scores with breastmilk micronutrient levels to further examine the effect of maternal micronutrient inadequacies on infants. 

Results from this preliminary analysis indicate that Hispanic, postpartum women are at risk of micronutrient inadequacy for some important micronutrients. These findings underline the importance of equitable and adequate access to nutritious foods particularly during a sensitive such as during the postpartum period. This also suggests that micronutrient intake should be considered when developing health promoting policies for Hispanic women during the postpartum period such as specific interventions to increase consumption of these nutrients through dietary supplementation or foods that are rich in these dietary components (i.e., animal protein, dairy, whole grains, nuts, legumes, fruits, and leafy green vegetables). Nonetheless, additional studies are needed to further understand the effects of micronutrients and micronutrient inadequacy on maternal and infant health. 

## Figures and Tables

**Table 1:** The mean and 25th–75th percentiles of usual intake from 1 to 6 months postpartum and percent of women below the estimated average requirement (EAR) or adequate intake (AI) after adjusting for timepoint and day of the week recall was performed. Usual intake was estimated after adjusting for within-person variation using the National Cancer Institute (NCI) method. ^A^ EAR and adequate intake (AI) values come from the National Institutes of Health (NIH) for lactating women 19–50 years old, *n* = 188, d = day.

**Nutrient**	**EAR [AI] ^A^**	**Adjusted Mean [25th–75th]**	**% < EAR [AI]**
Vitamins			
Niacin (mg/d)	13	32.8 [26.4–38.3]	0.3%
Vitamin B12 (µg/d)	2.4	9.7 [6.1–12.1]	0.9%
Riboflavin (mg/d)	1.3	2.9 [2.1–3.4]	2.7%
Thiamin (mg/d)	1.2	2.5 [1.9–3.0]	2.7%
Vitamin B6 (mg/d)	1.7	3.6 [2.6–4.3]	4.3%
Vitamin C (mg/d)	100	146.7 [104.2–181.0]	22.4%
Vitamin D (µg/d)	10	11.8 [7.6–14.9]	43.8%
Vitamin K (µg/d)	[90]	78.9 [60.7–92.8]	[71.8%]
Pantothenic Acid (mg/d)	[7]	5.6 [4.2–6.6]	[80.7%]
Folate (µg/d)	450	355.6 [296.7–405.0]	87.0%
Vitamin A (µg/d)	900	629.2 [509.1–732.5]	93.4%
Vitamin E (IU/d)	23.9	12.9 [8.8–15.7]	95%
Minerals			
Phosphorus (mg/d)	580	1364.4 [1165.2–1533.0]	0%
Iron (mg/d)	6.5	36.2 [23.5–44.5]	0.1%
Selenium (µg/d)	59	112.5 [95.1–127.1]	0.3%
Sodium (mg/d)	1500	2687.2 [2245.8–3053.0]	0.7%
Zinc (mg/d)	10.4	22.8 [15.4–27.9]	6.0%
Magnesium (mg/d)	255	306.4 [265.5–341.5]	18.8%
Calcium (mg/d)	800	1015.8 [780.5–1195.8]	27.6%
Manganese (mg/d)	[2.6]	2.8 [2.3–3.2]	[41.3%]
Copper (mg/d)	1.0	1.1 [0.9–1.2]	42.5%
Potassium (mg/d)	[2500]	2469.7 [2099.2–2781.8]	[56.5%]

## Data Availability

The data that support the findings of this study are available on request from the corresponding author. The data are not publicly available due to privacy and ethical restrictions.

## Supplementary Materials

The following are available online at https://www.mdpi.com/article/10.3390/nu13093252/s1, Supplemental Table S1: Average Adjusted Intake and Percent of Hispanic Women Not Meeting the Estimated Average Requirement or Adequate Intake Values for Vitamins and Minerals at 1 and 6 months Postpartum.
