# Supplementary material for: Risk of Micronutrient Inadequacy among Hispanic, Lactating Mothers: Preliminary Evidence from the Southern California Mother’s Milk Study
Source: Nutrients. 2021 Sep 18;13(9):3252. doi: 10.3390/nu13093252 (PMC8465791; doi:10.3390/nu13093252)
Supplement: Supplementary file 1 [file nutrients-13-03252-s001.zip › nutrients-1357814-supplementary.pdf]

Scheme 1. Average Adjusted Intake and Percent of Hispanic Women Not Meeting the Estimated Average Requirement or Adequate Intake Values for Vitamins and Minerals at 1- and 6-Months Postpartum.

Nutrient	EAR [AI][A]	1-Month Adjusted Mean [25th-75th]	% <EAR [AI] at 1-Month	6-Months Adjusted Mean [25th-75th]	% <EAR [AI] at 6-Months
Vitamin*s*					
Niacin (mg/d)	13	33.1 [24.6-40.7]	3	29.0 [22.0-34.6]	1.9
Vitamin B12 (µg/d)	2.4	8.8 [4.8-11.6]	6.9	8.4 [4.4-10.6]	5.8
Riboflavin (mg/d)	1.3	2.8 [1.8-3.5]	9.6	2.4 [1.5-2.9]	16.1
Thiamin (mg/d)	1.2	2.5 [1.6-3.1]	12	2.1 [1.3-2.6]	18.2
Vitamin B6 (mg/d)	1.7	3.5 [2.2-4.4]	12.7	2.9 [1.8-3.6]	20.8
Vitamin C (mg/d)	100	159.2 [102.2-203.9]	23.9	86.1 [47.0-113.0]	68.1
Vitamin D (µg/d)	10	11.0 [5.9-14.9]	51	10.2 [5.3-13.3]	58.9
Vitamin K (µg/d)	[90]	85.0 [62.6-101.5]	[63.6]	80.3 [59.0-95.4]	[69.5]
Pantothenic Acid (mg/d)	[7]	5.4 [4.0-6.5]	[81.7]	6.6 [4.3-8.1]	[64.6]
Folate (µg/d)	450	355.7 [296.2-405.0]	87	355.7 [296.2-405.0]	87
Vitamin A (µg/d)	900	608.5 [454.3-736.0]	90.4	576.2 [480.9-659.6]	98.2

Vitamin E (IU/d)	23.9	14.2 [8.6-17.6]	89.5	12.3 [8-15.1]	94.9
Minerals					
Phosphorus (mg/d)	580	1389.6 [1111.6-1610.5]	0.2	1375.4 [1131.6-1574.4]	0.1
Iron (mg/d)	6.5	38.3 [16.6-47.7]	3.2	20.7 [11.9-25.8]	4.2
Selenium (µg/d)	59	116.1 [94.0-133.8]	0.7	118.5 [96.3-136.5]	0.5
Sodium (mg/d)	1500	2498.0 [1996.2-2896.3]	4.4	3661.3 [2899.7-4260.6]	0.2
Zinc (mg/d)	10.4	24.0 [12.7-30.0]	16.2	15.9 [10.2-19.5]	26.4
Magnesium (mg/d)	255	321.0 [261.2-369.3]	22.3	284.2 [236.2-323.8]	36.6
Calcium (mg/d)	800	996.4 [716.2-1197.7]	35	846.7 [587.5-1026.9]	53
Manganese (mg/d)	[2.6]	2.9 [2.2-3.5]	[43.3]	2.4 [1.9-2.9]	[64]
Copper (mg/d)	1.0	1.1 [0.9-1.3]	38.6	1.0 [0.7-1.2]	60.4
Potassium (mg/d)	[2500]	2529.8 [1994.0-2949.4]	[54.4]	2368.1 [1957.8-2704.5]	[63.9]

Supplemental Table S1. Table shows the mean and 25th-75th percentiles of usual intake at 1- and 6-months postpartum and percent of women below the Estimated Average Requirement (EAR) or Adequate Intake (AI) after adjusting for the day of the week recall was performed. Usual intake was estimated after adjusting for within person variation using the National Cancer Institute (NCI) method. [A]EAR and Adequate Intake (AI) values come from the National Institutes of Health (NIH) for lactating women 19-50 years old, *n* = 188.
